# First person – Alice Yuen

**DOI:** 10.1242/bio.059445

**Published:** 2022-05-31

## Abstract

First Person is a series of interviews with the first authors of a selection of papers published in Biology Open, helping early-career researchers promote themselves alongside their papers. Alice Yuen is first author on ‘ A kinase translocation reporter reveals real-time dynamics of ERK activity in *Drosophila*’, published in BiO. Alice is a PhD student in the lab of Dr Marc Amoyel at the Department of Cell and Developmental Biology, University College London, London, United Kingdom, investigating the role of signalling pathways in influencing stem cell self-renewal and differentiation using clonal analysis and live imaging.


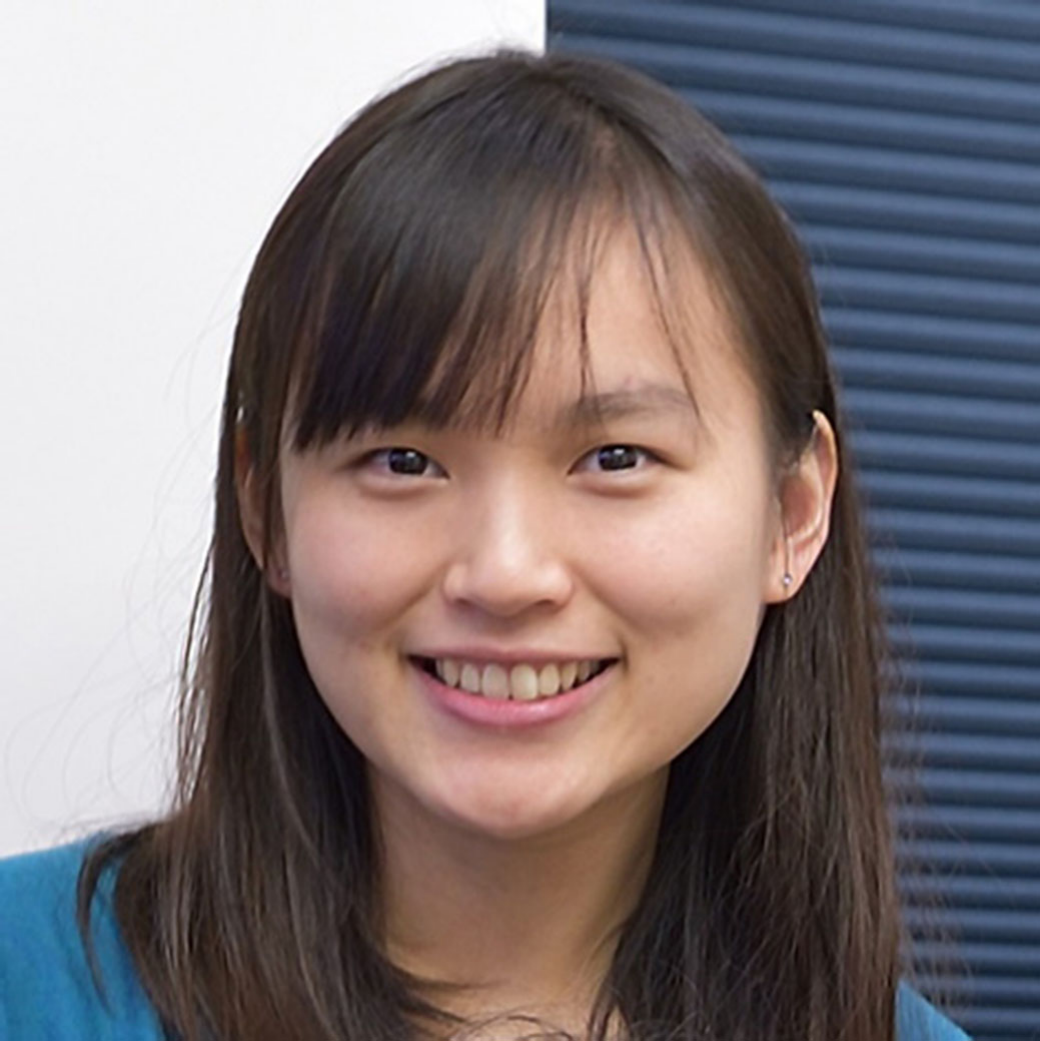


**Alice Yuen**

**What is your scientific background and the general focus of your lab?**

I was fortunate enough to be funded by the Nuffield Bursary Scheme while I was still at school to gain some insight into laboratory research in molecular neurobiology. Eager for more, I studied neuroscience at university where, funnily enough, I fell in love with developmental biology instead. I was amazed by the robustness of embryonic cells and their step-by-step transformation to generate the complexity of entire organisms. Fascinated with the question of how cells know what to do given their local and global environments, I joined Dr Marc Amoyel's lab for my PhD. Our lab studies how stem cells in the *Drosophila* testis compete with each other to remain in the niche. Part of our work focuses on the role of signalling in influencing a stem cell's decision to self-renew or differentiate.

**How would you explain the main findings of your paper to non-scientific family and friends?**

Animals are complex organisms made up of more than one cell, which means that cells must be able to talk to each other and coordinate their activities from the point of embryo formation through to adult life. Miscommunication can lead to diseases such as cancer and developmental disorders. The fruit fly is widely used in research as a model to study these diseases, as a steppingstone to understanding how human cells work. To complement ongoing research, we are presenting a new tool that allows us to eavesdrop on these conversations in real-time as they take place in the fly. Our tool has enabled us to make two novel and unexpected observations when we made movies to capture the development of fly larvae. We are now very excited to share this promising tool with the rest of the research community so that everyone can hear this curious chatter too!

**What are the potential implications of these results for your field of research?**

What is great about projects like these is that developing tools has the potential to touch on a broad range of topics. We wanted to be able to study cell–cell communication in the context of stem cell competition, but we had to take a step back and figure out ways to measure this live *in vivo*. Our development of a genetically encoded, tissue-specific ERK sensor for the fly will thus be useful to study how cells talk to each other in a wide range of contexts, including development and disease.

**Figure:**
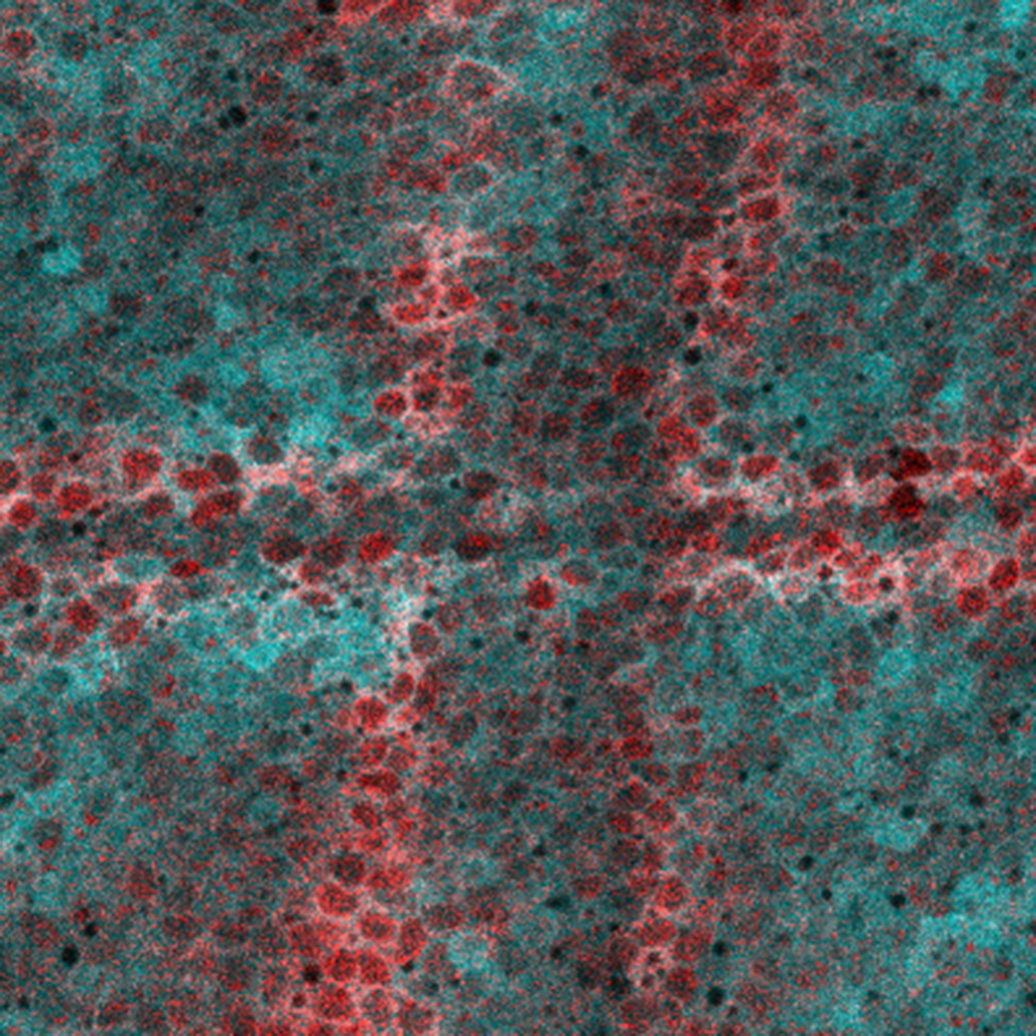
**Expression of our biosensor, ERK-KTR (blue), and immunohistochemistry for dpERK (red) in a developing wing imaginal disc.** Stripes of dpERK detected in the wing pouch will match the location of the future wing veins. ERK-KTR is predominantly nuclear-excluded in the prospective veins, reflecting high ERK activity.

**What has surprised you the most while conducting your research?**

I was most surprised by the difference between looking at signalling dynamics in live tissues as opposed to looking in fixed tissues. What appeared to be random ERK levels in snapshots of fixed cells turned out to be patterns of ERK activity correlated with specific cell behaviours during development. This highlighted for me the importance of following cells over time to really understand the relationship between signalling activity and cell behaviours.

“What appeared to be random ERK levels in snapshots of fixed cells turned out to be patterns of ERK activity correlated with specific cell behaviours during development.”

**What changes do you think could improve the professional lives of early-career scientists?**

It would be difficult to imagine being where I am now without the advice and support from my mentors. These channels of communication with those who can share their experiences are instrumental in helping early-career scientists prepare for the breadth of opportunities open to them. Greater emphasis should be placed on training, not just in practical and critical thinking skills, but also in mentorship – in learning how to both give and receive the support needed. Fostering better mentorship would encourage independence and creativity, and of course, can also be very rewarding. We should strive to support each other since scientific research is a concerted effort after all!

**What's next for you?**

I am about to finish my PhD and I am hoping to find a postdoctoral position where I can continue studying cell behaviours in the field of developmental biology.
